# Information Filtering in Sparse Online Systems: Recommendation via Semi-Local Diffusion

**DOI:** 10.1371/journal.pone.0079354

**Published:** 2013-11-18

**Authors:** Wei Zeng, An Zeng, Ming-Sheng Shang, Yi-Cheng Zhang

**Affiliations:** 1 Web Sciences Center, University of Electronic Science and Technology of China, Chengdu, People’s Republic of China; 2 Department of Physics, University of Fribourg, Fribourg, Switzerland; 3 Institute of Information Economy, Hangzhou Normal University, Hangzhou, People’s Republic of China; Universidad Carlos III de Madrid, Spain

## Abstract

With the rapid growth of the Internet and overwhelming amount of information and choices that people are confronted with, recommender systems have been developed to effectively support users’ decision-making process in the online systems. However, many recommendation algorithms suffer from the data sparsity problem, i.e. the user-object bipartite networks are so sparse that algorithms cannot accurately recommend objects for users. This data sparsity problem makes many well-known recommendation algorithms perform poorly. To solve the problem, we propose a recommendation algorithm based on the semi-local diffusion process on the user-object bipartite network. The simulation results on two sparse datasets, Amazon and Bookcross, show that our method significantly outperforms the state-of-the-art methods especially for those small-degree users. Two personalized semi-local diffusion methods are proposed which further improve the recommendation accuracy. Finally, our work indicates that sparse online systems are essentially different from the dense online systems, so it is necessary to reexamine former algorithms and conclusions based on dense data in sparse systems.

## Introduction

Owing to the rapid development of the Internet, people are confronted with abundant online contents, which makes it very time-consuming to select the needed information. This is often refereed as the information overload problem. In order to solve it, search engines and recommender systems are widely investigated and applied to real systems. The search engine returns the relevant contents based on the keywords given by users. Compared to the search engine, the recommender system provides personalized services for users by predicting the potential interests based on their historical choices.

Up to now, many recommendation algorithms have been proposed such as collaborative filtering (CF) [1]–[3], content-based analysis [4] and spectral analysis [5]. The matrix factorization algorithms have also been widely investigated by combining high scalability with predictive accuracy [6]–[7]. Recently, some physical processes, including mass diffusion [8], [9], heat conduction [10] and electric circuit analysis [11], have been applied to design recommendation algorithms. The hybridization of the mass diffusion and heat conduction algorithm is shown to effectively solve the diversity-accuracy dilemma in recommendation [12]. Based on these algorithms, many methods have been proposed to further enhance the recommendation diversity and solve the object cold-start problems. For example, the preferential diffusion [13], the biased heat conduction [14], network manipulation [15] and the item-oriented method [16] are shown to be able to largely improve the recommendation accuracy for small-degree objects. More recently, the long-term influence of the hybrid approach on network evolution has been studied [17].

One of the biggest challenges in recommender systems is the data sparsity problem. That is, the user activity data is too sparse for the recommender system to provide satisfactory recommendations. To solve such sparsity problem, the users’ social network is incorporated in the object recommendation. For instance, a random walk model based on both the trust network and user-object bipartite network was designed [18]. Based on the matrix factorization method, both the user trust network and friendship network can be fused in the object recommendation by regularization [19], [20]. Yang [21] proposed a factor-based random walk model to recommend both online services and friends to users. In addition, the users’ membership data (i.e. the social groups that online users joined) is considered and the results indicate that this social information is more valuable than friendship when used to enhance the recommendation accuracy of object [22].

However, the users’ social network is usually much sparser than the user-object network in most systems. More importantly, those users who have collected or purchased few objects might also be inactive in building their social relationships. Therefore, the compensation effect of social networks on the user-object bipartite networks is limited. In this paper, we propose an approach based on the semi-local diffusion process on the user-object bipartite network to solve the data sparsity problem. Our simulation results on two real datasets, Amazon and Bookcross, indicate that our method significantly outperforms the state-of-the-art methods especially for these small-degree users. Moreover, two personalized semi-local diffusion methods are proposed which further improve the accuracy.

## Data Sparsity Problem

An online commercial system can be usually represented by a bipartite network 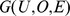, where 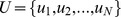, 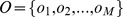 and 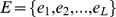 are the sets of users, objects and links, respectively. Denote by an adjacency matrix 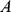, where the element 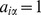 if user 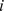 has collected item 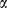, and 0 otherwise (throughout this paper we use Greek and Latin letters, respectively, for item- and user-related indices) [2], [23].

The hybrid method in ref. [12] takes into account both the mass diffusion [8] and the heat conduction [10] process. This method is shown to be able to provide not only accurate but also diverse recommendations for users when applied to dense datasets. Here, we argue that this hybrid method fails in sparse datasets. As an example, we test this hybrid method on two sparse datasets: Amazon (www.amazon.com) and Bookcross (www.bookcrossing.com). *Amazon.com* is a multinational e-commerce company and the world’s largest online retailer. The original data was collected from 28 July 2005 to 27 September 2005 [24]. During this period, there are 1,714,512 reviewers in total. The data contains 100,000 highest ranked reviewers and all reviews written by them. Some of the reviewers in the list didn’t give reviews during this period of time, so that in practice only 99,622 reviewers contributed. They wrote total 2,036,091 reviews on 645,056 products. Here, we select a random subset from the data. *Bookcrossing.com* is a book sharing web site where book lovers can exchange their books and experiences with each other. The original data has 278, 858 users and 1, 157, 112 ratings, referring to 271, 379 distinct ISBNs (objects) [25]. Invalid ISBNs were excluded from the dataset. The complete BookCrossing dataset is available online (http://www.informatik.uni-freiburg.de/~cziegler). The data in this paper is a random sample from the original data. Some basic statistics of these two datasets are presented in the Table 1. Each data is randomly divided into two parts: the training set (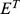) and the probe set (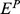). The training set contains 80% of the original links and the recommendation algorithm runs on it [26]. The rest of the links forms the probe set, which will be used to examine the recommendation performance.

**Table 1:** The statistics of Amazon and Bookcross datasets.

Dataset	#user	#objects	#links	sparsity
Amazon	50000	54,152	283,382	1035×10^−4^
Bookcross	21122	203,373	504,643	1.17×10^−4^

The sparsity is obtained by 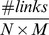, where 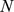 and 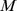 are the number of users and items, respcetively.

**Figure 1:**
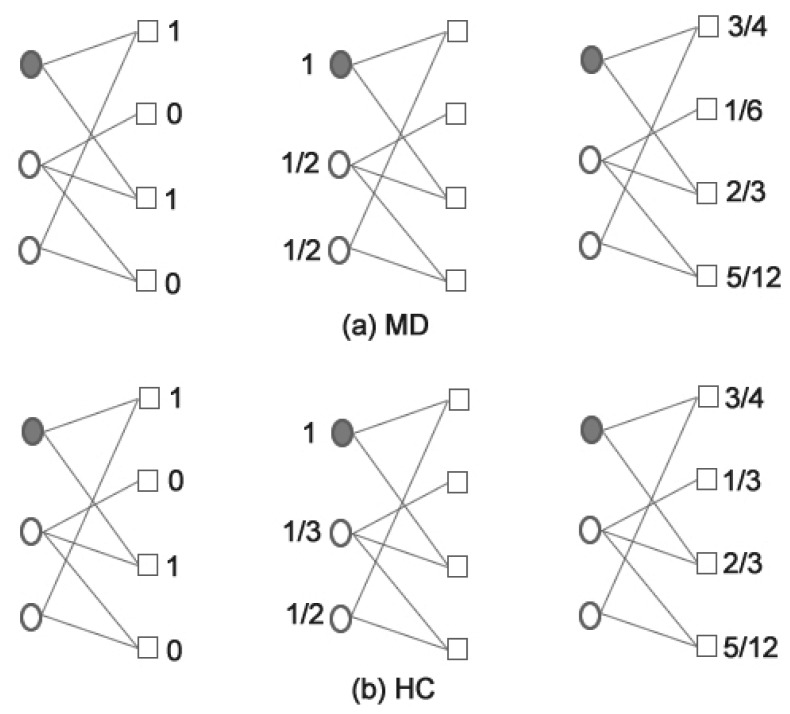
The Mass Diffusion (a) and Heat Conduction (b) algorithms at work on the bipartite user-object network. Users are shown as circles; objects are squares. The target user is indicated by the shaded circle.

When recommending objects for user 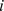, the hybrid method works by assigning each object collected by user 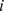 one unit of resource. The initial resources are denoted by the vector 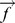 where 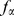 is the resource possessed by object 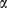. Then they will be redistributed via the transformation 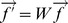, where
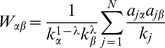
(1)is the redistribution matrix, with 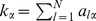 and 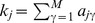 denoting the degree of object 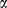 and user 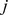, respectively. 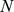 and 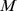 are the number of users and objects, respectively. 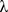 is a tunable parameter which adjusts the relative weight between the Mass Diffusion algorithm (short for MD, 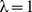) and Heat Conduction algorithm (short for HC, 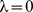). The illustration of MD and HC algorithms can be seen in Fig. 1(a) and (b), respectively. The resulting recommendation list of uncollected items is sorted according to 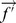 in descending order.

In order to measure the recommendation accuracy, we make use of the ranking score (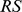). Specifically, 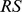 measures whether the ordering of the items in the recommendation list matches the users’ real preference. For a target user 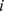, all her/his uncollected items will be ranked according to their predictive scores in the descending way by the recommender system. Given 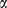 is an object selected by user i in the probe set, 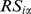 is the rank of 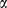 in 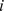
’s recommendation list divided by the total number of uncollected items by user 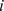. The smaller the 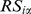, the better the recommendation, the items in the probe set being ranked higher. The mean value of the 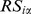 over all the user-item relations in the probe set can be used to evaluate the recommendation accuracy as
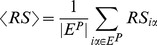
(2)


The smaller the value of 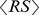, the higher the recommendation accuracy.

**Figure 2:**
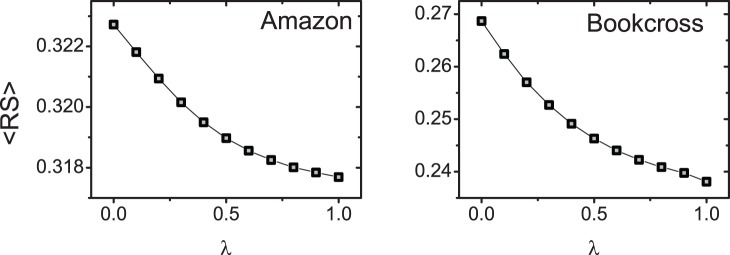
The ranking score of the hybrid method on Amazon and Bookcross. 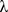
 is used to tune the contribution of the heat conduction and the mass diffusion process. When 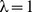, the hybrid method gives the pure mass diffusion method and 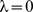 it degenerates to pure heat conduction method (more details about the hybrid method can be found in [12]). Each data point is obtained by averaging over ten runs, each of which has an independently random division of training set and probe set.

In ref. [12], 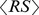 can achieve an optimal value when adjusting the parameter 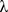 of the hybrid recommendation method. However, when applied to the sparse data mentioned above, 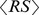 changes monotonously with 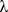, as presented in Fig. 2. In other words, the recommendation accuracy cannot be improved by taking into account the heat conduction process in the mass diffusion method.

**Figure 3:**
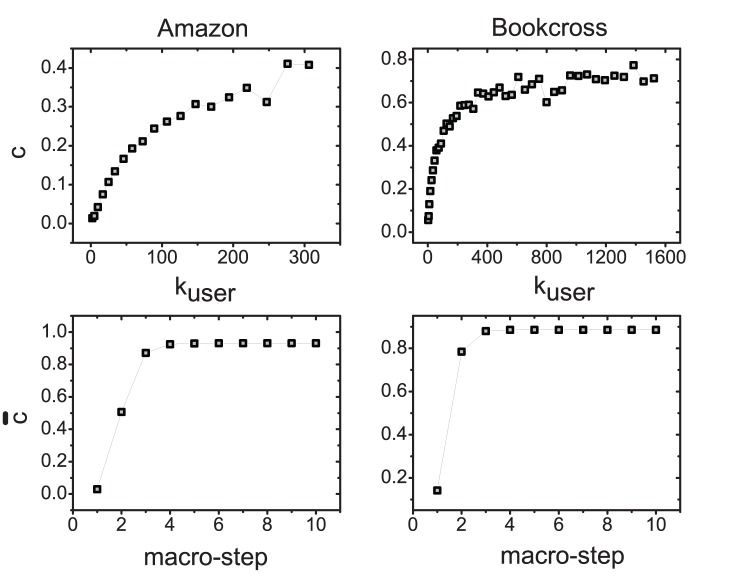
The coverage 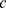 and avgerage coverage 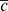 in Amazon and Bookcross. The top two subfigures plot the dependance of the coverage 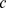 on the user degree. For a given 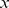, its corresponding 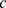 is obtained by averaging all the users whose degrees are in the range of 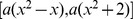, where 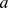 is chosen as 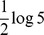 [13]. The bottom two subfigures plot the relations between the average coverage 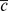 and the macro-step.

To understand the reason, we introduce a concept called *coverage*, 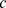. As shown in Fig. 1, the diffusion-based algorithms are based on 3 steps. Given the diffusion starting from user 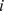, we denote the objects whose received resources are larger than 0 after 3 diffusion steps as covered objects. Then the coverage 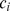 is defined as the number of covered objects divided by the number of unselected objects by user 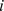. Actually, this definition has been used before in [27]. The larger 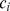 is, the more objects will receive resources in the Hybrid method. The average coverage 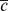 over all users are 0.0301 for Amazon and 0.1413 for Bookcross, respectively. In other words, most objects will receive 0 resource if we choose the Hybrid algorithm. Note that the hybridization [12] only changes the amount of resource of the covered objects. The resource of the uncovered objects will stay 0 under all hybrid parameters. Since the coverage dominates the recommendation accuracy in sparse data, the hybrid method cannot improve the recommendation accuracy as shown in Fig. 2. Moreover, we show the relationship between the user degree and the coverage 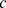 in the top subfigures of Fig. 3. The coverage nonlinearly increases with user degree, which leads to an even more serious user cold-start problem. In next section, we will propose a semi-local diffusion method to increase the diffusion coverage and break the tie among these items with 0 resource.

## Algorithm and Metrics

Our semi-local diffusion method will be directly built on the mass diffusion method [8]. The MD method is simply the case when 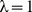 in the hybrid method. Given a target user 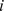, the first step of MD is to allocate one unit resource to each of 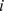
’s collected items. Due to the bipartite structure, it takes two diffusion steps for the resource to get back to the item side. For convenience, we denote every 2 steps after the 1-step as one macro-step (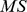 for short) of diffusion. The original 3-step diffusion is combined by the first ordinary step (the initial resources allocating process) and 1 macro-step diffusion. As discussed above, the original 3-step diffusion method suffers from the data sparsity problem since most objects’ resources are 0. To solve this problem, we let the resources diffuse on the bipartite network more than one macro-step. The initial resources are denoted by the vector 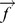. After one macro-step, items’ resource can be expressed as 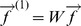, where 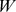 is the resource redistribution matrix for mass diffusion algorithm (with 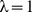 in equation 1). Likewise, we can calculate items’ resource after 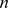 macro-steps of diffusion as 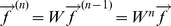. To recommend objects to user 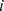, one can sort the 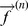 in descending order and those objects with most resources will be recommended. Since the algorithm above uses less than global information but a bit more than pure local information, we call this method as Semi-Local Diffusion (SLD) recommendation method.

In previous section, we used the ranking score to measure the recommendation accuracy. Since real users usually consider only the top part of the recommendation list, a more practical measure should take into account the number of a user’s hidden links contained in the top-L places. Therefore, we use another recommendation accuracy measure called “Recall”. As discussed above, the real data is first divided into two parts: training set and probe set. For each user 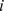, he/she may have certain number of links (corresponding to objects) in the probe set, we denote it as 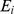. After the recommendation list (with length 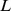) is generated for user 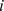, we will calculate 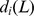 as the number of his/her probe set objects which appear in the recommendation list. The Recall of this user is defined as
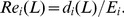
(3)


The Recall of the whole system is defined as
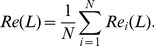
(4)


A higher Recall value indicates a higher accuracy of recommendation.

## Results

**Figure 4:**
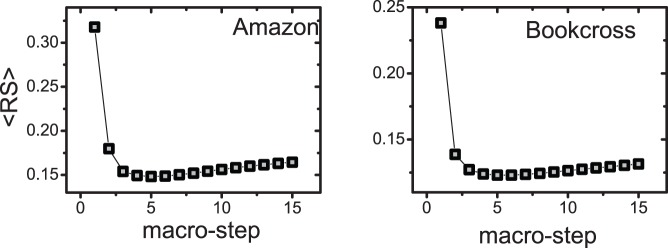
The ranking score 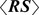 of the semi-local diffusion method in Amazon and Bookcross. For both datasets, we obtain the lowest ranking score when the macro diffusion step is 5. Each data point is obtained by averaging over ten runs, each of which has an independently random division of training set and probe set.

If we let the objects’ resources diffuse on the bipartite network for multiple macro-steps, more objects will be covered. We plot the relations between the average coverage 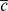 and the macro-step in the bottom two subfigures of Fig. 3. As one can see, the average coverage 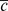 increased quickly with the macro-step. Therefore, more objects in the probe set may receive resource in the diffusion and have higher rank accordingly. The relation between the overall 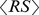 and the number of macro-steps is presented in Fig. 4. If *macro-step = 1*, the method degenerates to the standard Mass diffusion method. From the figure, one can see that 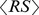 is improved significantly by the SLD method and the optimal macro-step is 5 in both datasets. If the macro-step is more than 5, the ranking score gets worse but still much better than that of the original MD method. We actually test the other diffusion methods based on one macro-step [13], [14], [16], and the results show that 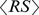 of these methods are similar to MD in sparse networks. The parameters in these methods only slightly influence the results. A network manipulation method was proposed to solve the object cold-start problem by adding some virtual links to the network [15]. However, this method is also found less effective than the SLD method. This is because the virtual links inevitably contain some noise and the recommendation based on sparse data is very sensitive to the noise.

**Figure 5:**
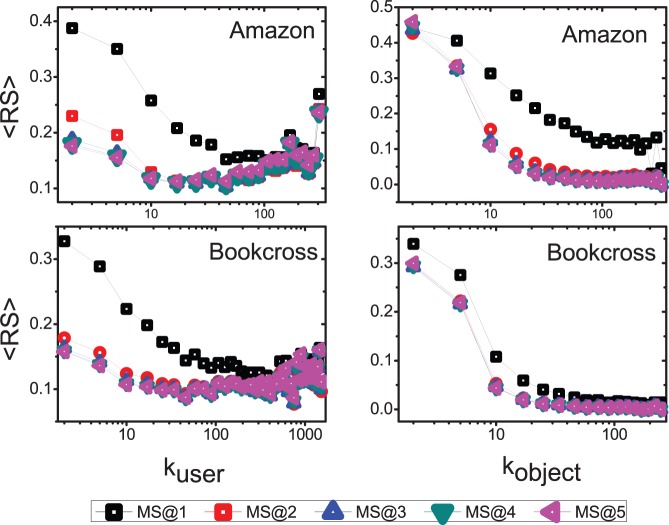
Dependence of the ranking score 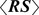 on user degree and object degree. The 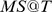 means that 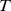 is the macro-step of the diffusion. For a given 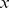, its corresponding 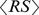 is obtained by averaging all the users (or objects) whose degrees are in the range of 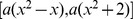, where 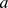 is chosen as 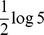 [13]. Each data point is obtained by one run since the degree of a user and an item may change for different dataset divisions.

Additionally, we report the dependence of 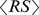 on the user degree and object degree in Fig. 5. The left two figures of Fig. 5 give the relationship between the user degree and 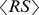. One can see that 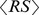 of small-degree users who have collected few objects are improved greatly since these users’ *coverage* of objects are increased significantly by the SLD. The right two figures of Fig. 5 show the relationship between the object degree and 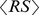. It can be seen that the SLD can improve 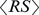 of both the small-degree and large-degree objects.

**Figure 6:**
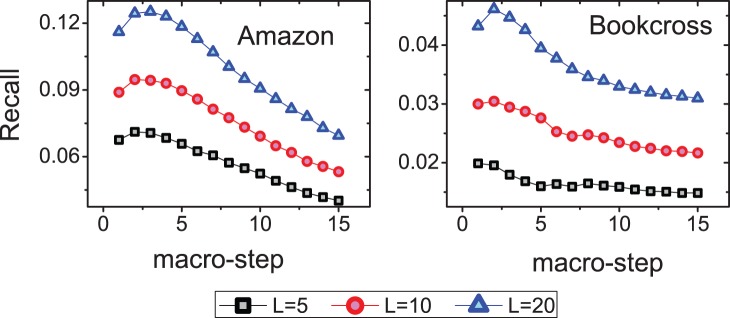
The Recall of the semi-local diffusion method in Amazon and Bookcross. For both datasets, we obtain the best performance when the macro-step of the diffusion is 2. Each data point is obtained by averaging over ten runs, each of which has an independently random division of training set and probe set.

**Figure 7:**
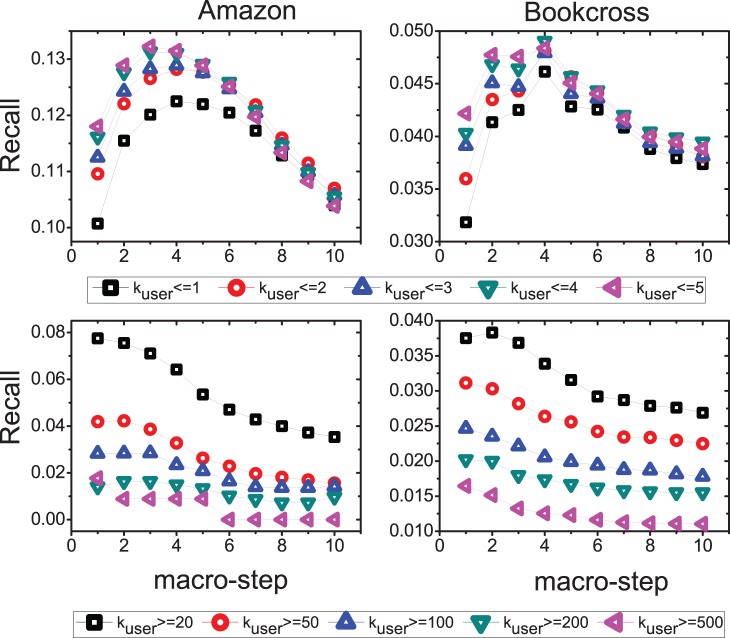
Dependence of *Recall* on the diffusion macro-step. The recommendation list length 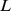 is set to 20. 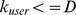 means that we only consider the users whose degree is no larger than 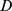. Each data point is obtained by one run since the degree of a user and an item may change for different dataset divisions.

**Figure 8:**
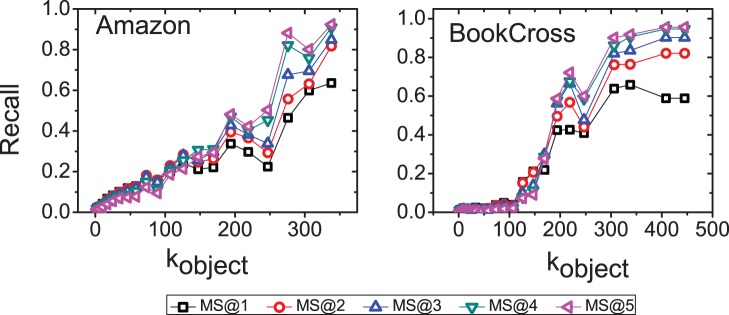
The relationship between object degree and *Recall*. The recommendation list length 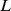 is set to 20. The 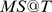 means that 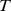 is the macro-step of the diffusion. Each data point is obtained by one run since the degree of a user and an item may change for different dataset divisions.

Another interesting question is whether the accuracy of top-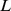 recommendation list will be improved the same as the ranking score by the SLD. The relation between the Recall and the number of macro-steps is presented in Fig. 6. For both datasets, we get the best performance when the macro-step is 2. However, when the macro-step exceeds 2, the Recall of both datasets starts to decrease. To uncover the reason, we study in detail the relationship between the top-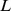 accuracy and user degree and object degree, respectively. Since Recall is defined based on users, it can be naturally used to measure the recommendation accuracy of the users with the same degree. When applied to objects, we define the object Recall as: 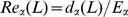 where 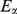 is the number of users who selected object 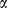 in the probe set, and 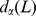 is the number of times that 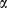 appears in these 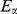 users’ recommendation lists. The Recall of the objects with the same degree is obtained by simply averaging 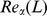 of these objects. The top two subfigures of Fig. 7 show 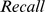 of the users whose degrees are no larger than 5 . It can be seen that the accuracy of top-20 recommendation lists of those inactive users are improved considerably by the SLD if the macro-step of diffusion is less than 5. The best macro-step is 3 for Amazon and 4 for Bookcross, respectively. If the macro-step of diffusion exceeds 5, the 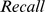 of those users starts to decrease. The bottom two subfigures of Fig. 7 give the 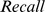 of the users whose degrees are no smaller than 20. It shows that the 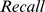 decreases monotonously with macro-step. In addition, we plot the relationship between 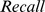 and the object degree in Fig. 8. It shows that the SLD method tends to improve the 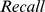 of large-degree objects. Generally speaking, small degree users incline to select popular items [28]. However, since the small degree users only have limit number of links, the original 3-step diffusion cannot reach the relevant popular items for them. On the other hand, the SLD method effectively increases the diffusion coverage and discover the most relevant popular items for these small degree users. This is of great importance from practical point of view since these new/inactive users are very sensitive to the quality of recommendation and poor quality may lead to losing them from the website.

Our result above shows that the high order diffusion resources may play different role in the recommendation for users and objects with different degrees. Therefore, the information of the high order diffusion should be used in a personalized way. Accordingly, we propose two extended recommendation methods: the user-based semi-local diffusion method (U-SLD for short) and the object-based semi-local diffusion method (O-SLD for short). We denote 
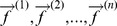
 as the final resource vectors after *1, 2, …, n* macro-steps of diffusion, respectively. 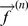 can be easily calculated by 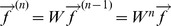. Given the target user 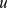, the user-based semi-local diffusion method is to combine these 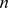 resource vectors based on 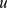
’s degree. Mathematically, the final score of object 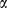 reads
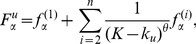
(5)where 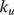 is 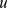
’s degree, 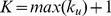 and 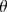 is a free parameter to tune the weight of 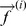 (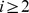) based on 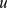
’s degree. If 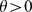, the second term will play a more significant role when recommending objects for large-degree users, and vice versa.

In the sparse dataset, the coverage of 3-step diffusion is very low. Even some popular items cannot be effectively reached by users. The object-based semi-local diffusion method accumulates those resources based on the object degree. The final score of object 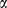 computed by this method is
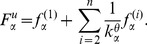
(6)


**Figure 9:**
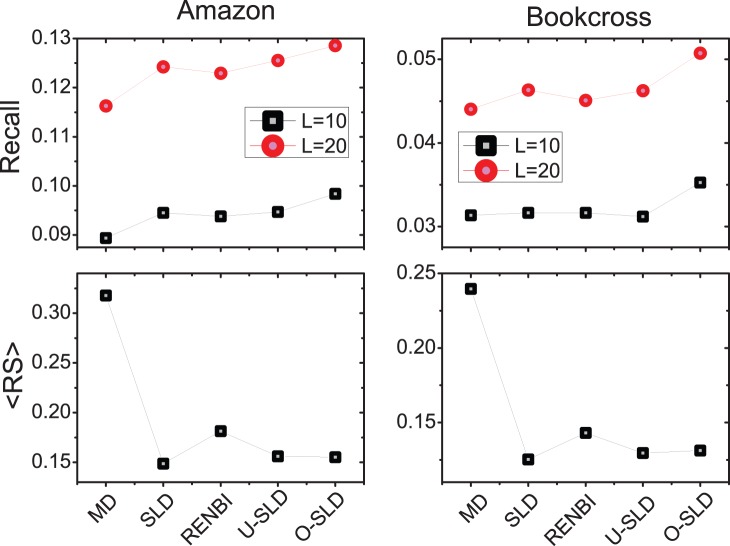
The accuracy comparison of different algorithms. Each data point is obtained by averaging over ten runs, each of which has an independently random division of training set and probe set.

If 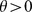, the second term will play a more significant role in calculating the score for small-degree items, and vice versa. We sort the vector 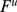 in descending order and those objects with highest scores will be recommended to 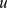. The results on Amazon and Bookcross are reported in Fig. 9 and the optimal parameters 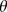 of algorithms discussed above are presented in Table 2. In order to balance the improvement on *ranking score* and *Recall*, we set 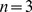 in both U-SLD and O-SLD.

**Table 2:** The optimal parameter defined in algorithms for *Recall* and *Ranking score*.

Amazon					
		SLD-T	RENBI	U-SLD	O-SLD
Recall	T	2	–	–	–
	*θ*	–	2	−0.9	−0.3
Ranking score	T	5	–	–	–
	*θ*	–	2	−1	−0.5

Bookcross					
		SLD-T	RENBI	U-SLD	O-SLD-
Recall	T	2	–	–	–
	*θ*	–	1.0	−0.6	−0.2
Ranking score	T	5	–	–	–
	*θ*	–	2	−1	−0.7

Actually, similar idea has been applied to eliminate the redundant correlations in dense datasets [29]. The method in [29] is called RENBI method and defined as
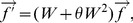
(7)where the elements of matrix 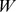 are defined by Eq. 1 with 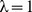, 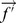 and 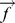 is the final resource vector and the initial resource vector, respectively, and 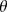 is a free parameter. In [29], the authors focus on improving the accuracy and diversity of recommendation by eliminating the redundant information and they find that the optimal 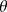 defined in Eq. 7 is negative. However, the information of high order diffusion is not redundant any more in sparse dataset. Moreover, the RENBI method is not personalized since the weight of high order diffusion resources is the same for all users. We will compare the U-SLD and O-SLD methods to the RENBI method.

The top subfigures of Fig. 9 show the results of *Recall* in Amazon and Bookcross. Clearly, the Recall of SLD is much higher than that of MD in both datasets. This is because the recommendation accuracy of small-degree users is significantly improved by SLD. Moreover, the RENBI method is also better than the MD method, but it is worse than the SLD. From the Table 2, we can also see that the optimal 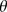 in Eq. 7 are 0.9 for Amazon and 0.7 for Bookcross, respectively. This is different from the result in ref [29] where the method is tested in dense data and the optimal 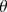 is found to be negative. Our results indicate that the information of high order diffusion is in fact not redundant information in the sparse data. Both the U-SLD and O-SLD methods are better than the RENBI method in Recall. The improvement is due to the personalized use of the high order diffusion information. Finally, we can see that the O-SLD achieves the best *Recall* among these methods and the optimal 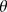 defined in Eq. 6 is negative in both datasets from Table 2. That is to say, the information of high order diffusion should be considered more on the large degree items than small degree items. This is because small degree users inclines to select the popular items while these items cannot be effectively reached by one macro-step diffusion. Note that once those small degree users have selected many objects, we could then recommend diverse objects to them.

The bottom subfigures of Fig. 9 show the results of *ranking score* in Amazon and Bookcross. One can see that the ranking score of SLD method is much lower than that of MD. From the Table 2, it is shown that the optimal diffusion step is 5 in both datasets. RENBI also achieves a considerable improvement in ranking score compared to MD, but its ranking score is higher than that of SLD. The optimal 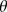 of RENBI is also positive in both datasets. This supports again that the high order diffusion information is actually useful in enhancing the recommendation accuracy in sparse data. Although the ranking score of U-SLD and O-SLD method are slightly higher than the SLD method, these two methods enjoy a much better ranking score than RENBI. Taking together the results of ranking score and Recall, O-SLD seems to be the best recommendation algorithm in sparse data based on these training sets. It provides not only a good ranking of users’ unselected objects but also an accurate top-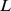 recommendation list.

## Discussion

The data sparsity problem is one of the biggest challenges in recommender systems. There are a large number of online users and objects with very few connections, which leads to the poor performance of many well-known recommendation algorithms. However, the data sparsity problem has not yet been systematically studied and not yet well addressed. Take the hybrid method [12] for example, one cannot get an improved recommendation accuracy when combining the mass diffusion and heat conduction algorithms. As a matter of fact, the data of most real online systems is much sparser than the data used in this paper. Therefore, solving the data sparsity problem is of great significance from the practical point of view.

In this paper, we propose a semi-local diffusion (SLD) method to solve the data sparsity problem in recommender systems. The results on two real online datasets indicate that our method significantly outperforms other well-known algorithms. Two personalized semi-local diffusion methods are also proposed which further improve the accuracy. Our analysis shows that the recommendation accuracy of small-degree users is greatly improved by the SLD method. In practical use, it can largely improve the experience of the new comers, so that more users will be attracted by the web site.

Finally, we remark that sparse online system are essentially different from the dense online system. Actually, most diffusion-based recommendation algorithms can be decomposed into two steps. The first step is to find all the relevant objects to the target user (i.e. objects covered by diffusion) and the second one is to rank these relevant objects. In the dense systems, the number of relevant objects is generally very large. Therefore, an effective recommendation algorithm in these systems should provide an accurate ranking of these relevant objects. However, the relevant objects in sparse systems are usually very limited and the objects the target user interested in might not be included in her/his relevant objects after 3-step diffusion. Accordingly, a more important issue for the recommendation algorithm in these systems should properly enlarge the number of relevant objects. Since the main task in designing recommendation algorithms in these two systems are different, all the algorithms and conclusions based on dense data should be rechecked in sparse data.
